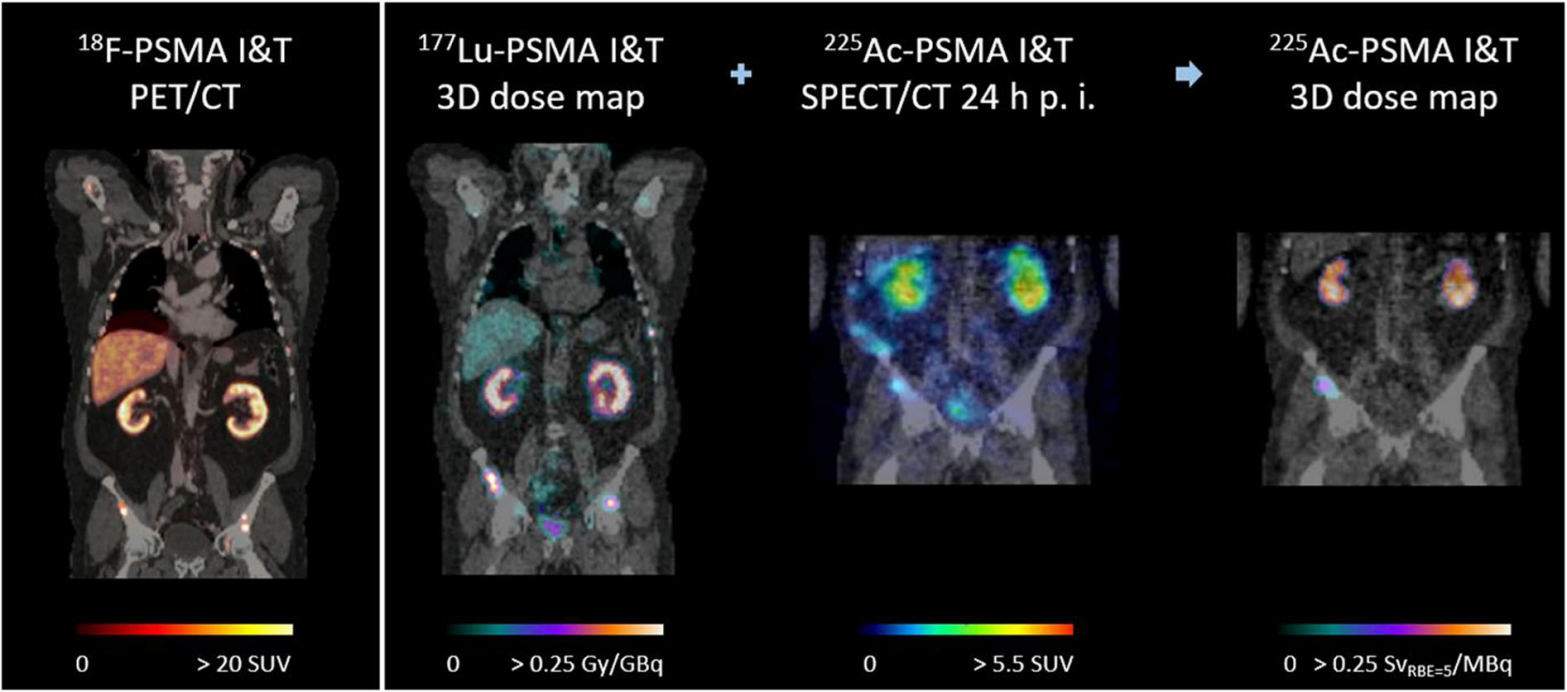# Image-based dosimetry for ^225^Ac-PSMA-I&T therapy using quantitative SPECT

**DOI:** 10.1007/s00259-020-05024-1

**Published:** 2020-09-21

**Authors:** A. Gosewisch, M. Schleske, F. J. Gildehaus, I. Berg, L. Kaiser, J. Brosch, P. Bartenstein, A. Todica, H. Ilhan, G. Böning

**Affiliations:** Department of Nuclear Medicine, University Hospital, LMU Munich, Munich, Germany

Targeted alpha therapy (TAT) using ^225^Ac-PSMA ligands is a promising therapy option for advanced metastatic castration-resistant prostate cancer (mCRPC) [1]. The ^225^Ac decay chain shows a noticeable gamma emission (440 keV, 25.9%; 218 keV, 11.4%). However, recommended low therapeutic activities (4–8 MBq) limit the clinical applicability of SPECT [2], although initial attempts for ^225^Ac imaging exist [3, 4]. Particularly quantitative SPECT is a vital tool to assess dosimetry and therapy response. While the 218-keV-peak is characterized by a lower branching ratio and a higher scatter fraction, SPECT imaging of high-energy gammas such as 440 keV causes a complex detector point spread function (PSF) [5].

In this study, we would like to demonstrate the general feasibility of image-based dosimetry for ^225^Ac radionuclide therapy using quantitative ^225^Ac SPECT. For a mCRPC patient (65 years), imaging of the abdomen was performed 24 h p. i. of 8.1 MBq ^225^Ac-PSMA-I&T on a Siemens Symbia Intevo T16 SPECT/CT (440 keV (width, 20%), lower adjacent window (width, 10%), HEGP collimator, 16 projections/head, 128 × 128 pixel, 210 s/projection). Reconstruction was carried out via a MAP algorithm (30i1s) [6], including CT-based attenuation and dual-energy-window scatter correction and a simulated distance-dependent 2D PSF model (SIMIND). Final absorbed dose assessment was performed by combining the single ^225^Ac image with the effective half-life information determined from a previous ^177^Lu-PSMA-I&T imaging sequence [7]. This resulted in an absorbed dose of 0.18 and 0.17 Sv_RBE = 5_/MBq for the left and right kidney, respectively, compared with 0.27 and 0.24 Gy/GBq for the preceding ^177^Lu cycle (6.2 GBq). A comparison with the pre-therapy ^18^F-PSMA-I&T PET/CT demonstrates that ^225^Ac SPECT imaging for this patient was able to locate a small lesion in the right hip. The ^225^Ac-absorbed dose was determined as 0.26 Sv_RBE = 5_/MBq, compared with 0.35 Gy/GBq for ^177^Lu-PSMA-I&T.

Our analysis demonstrates the feasibility of dosimetry for ^225^Ac-PSMA-I&T, which provides further insights into theranostic approaches using TAT in mCRPC patients.